# ACUTE RADIATING LOW BACK PAIN IMPACT ON ROUTINE AND FUNCTION OF THE BRAZILIAN POPULATION: A CROSS-SECTIONAL STUDY

**DOI:** 10.1590/1413-785220233105e266200

**Published:** 2023-10-23

**Authors:** GUILHERME HENRIQUE PORCEBAN, ALEXANDRE FELIPE FRANÇA FILHO, RENATO HIROSHI SALVIONI UETA, DAVID DEL CURTO, EDUARDO BARROS PUERTAS, MARCEL JUN SUGAWARA TAMAOKI

**Affiliations:** 1 Universidade Federal de São Paulo, Escola Paulista de Medicina, Departamento de Ortopedia e Traumatologia, São Paulo, SP, Brazil.

**Keywords:** Sciatica, Low Back Pain, Radiculopathy, Ciática, Dor Lombar, Radiculopatia

## Abstract

Acute radiating low back pain is a frequently occurring clinical condition among the population, and it represents a significant portion of urgent care in public health services.

**Objective:**

Consider the clinical characteristics, demographics, as well as the intensity of the pain, discomfort, and dysfunction of patients who show a clinical diagnosis that is compatible with acute radicular pain, new or reoccurring after an asymptomatic period.

**Methods:**

Patients that display a clinical diagnosis that is compatible with acute sciatic nerve pain, with the beginning of it starting within three months, without previous history of a similar occurrence, were seen in an orthopedic health clinic from July 2020 to January 2021.

**Results:**

A total of 42 patients were seen with a compatible diagnosis, which represents 1.4% of all medical visits. To the best of our knowledge, no studies have considered the clinical and demographic characteristics of patients with acute radicular pain in the Brazilian population. This study has found a mean value on the disfunction index that is greater than what is suggested by the current literature.

**Conclusion:**

About 30% of individuals showed functional involvement that was considered crippling, which presented a stronger association with individuals with the presence of motor deficits, intensity of radiating pain, and professional inactivity. **Level of Evidence IV, Cross-Sectional Study.**

## RESUMO

A lombociatalgia aguda é uma condição clínica bastante frequente na população e representa uma porção expressiva dos atendimentos de urgência nos serviços de saúde pública.

**Objetivo:**

Avaliar as características clínicas e demográficas, bem como a intensidade da dor e da disfunção de pacientes com quadro clínico compatível com dor radicular aguda, inédita ou recorrente após período assintomático.

**Métodos:**

Consideraram-se pacientes com quadro clínico compatível com dor ciática aguda que tenha se iniciado em até três meses, sem história prévia de episódio semelhante e que foram atendidos em uma unidade de pronto atendimento ortopédico entre julho de 2020 e janeiro de 2021.

**Resultados:**

Foram atendidos 42 pacientes com quadro clínico compatível, representando 1,4% do total de atendimentos realizados no serviço. Não há relatos de estudos que buscaram avaliar as características clínicas e demográficas dos pacientes com dor radicular aguda na população brasileira. Neste estudo, contudo, encontrou-se um elevado valor médio no índice de disfunção em comparação com a literatura atual.

**Conclusão:**

Cerca de 30% dos indivíduos apresentaram acometimento funcional considerado incapacitante, sendo que a presença de déficits motores, a intensidade de dor irradiada e a inatividade trabalhista foram estatisticamente maiores nesse grupo em relação aos demais. ***Nível de Evidência IV, Estudo Transversal.***

## INTRODUCTION

Acute radiating low back pain, also known as sciatic pain or sciatica, is a very common clinical condition in the population and accounts for a significant portion of emergency care visits in public healthcare services.^1^ When associated with deficits in strength and sensitivity in a specific dermatome or myotome of the lower limbs, it is referred to as radiculopathy. The most common etiology is discogenic causes, such as lumbar disc herniation,^2^ however, degenerative, joint-related, neoplastic, and infectious causes can also present with these symptoms.^3^

The incidence and prevalence of sciatica is still controversial, with wide variation among studies available in the literature. An epidemiological review from 2008^4^ identified that the prevalence of symptoms consistent with radicular pain ranged from 1.6% to 43%, with annual prevalence varying in the literature from 2.2% to 34%.^5^ On the other hand, robust scientific evidence has pointed to an association between the incidence of this condition and the patient’s age, being rare before the age of 20 and more often around the age of 50, decreasing afterwards.^6^

The natural history of acute radicular pain (onset within the last three months) is mostly benign, with approximately 70% of patients experiencing significant improvement within four weeks, and 60% of these individuals returning to work within that period^7^ after clinical treatment involving rest, analgesia, and physical rehabilitation. Despite this, acute sciatica presents great direct and indirect socioeconomic impacts due to its high prevalence and the fact that it mostly affects the most economically active population.^8^

We have not found any studies investigating the epidemiology of acute radicular pain in the Brazilian social context. This study aims to assess the clinical and demographic features, as well as pain intensity and dysfunction in patients with clinical profiles consistent with acute radicular pain, whether it is a first-time experience or a recurrence after a symptom-free period. The study was conducted at an orthopedic emergency unit that is reference in the Brazilian public health system.

## METHODS

### Study design

This cross-sectional observational study was conducted in the orthopedic emergency unit of a quaternary care hospital that serves as a reference facility within the Brazilian Unified Health System, namely the Sao Paulo Hospital of the Universidade Federal de Sao Paulo.

This research is an extension of another umbrella project being conducted by the same group of researchers, which has received approval from the Research Ethics Committee of the institution under reference number 4.232.193 (CAAE: 32486420.7.0000.5505).

### Participants and procedures

Patients exhibiting clinical symptoms consistent with acute sciatic pain-radiating low back pain to lower limbs accompanied by positive results in femoral stretch tests-within the past three months, without any history of a similar episode, and presenting to an orthopedic emergency department from July 2020 to January 2021 were included for the evaluation by spine group. Eligible participants received information about the study procedures and signed an informed consent form.

Patients with symptoms present for over three months prior to the study, exhibiting consistent clinical history and ongoing symptoms, as well as those with a history of spinal surgeries, infections, or trauma/fractures, were excluded from the study. Additionally, the presence of comorbidities or personal history that contradicts the use of oral corticosteroids, which is investigated by this umbrella project, was also considered as a criterion for exclusion.

During the initial interview, the research team physicians collected demographic/social and clinical data. They also applied the translated and adapted Brazilian Portuguese version of the Oswestry Disability Index (ODI) and the Visual Analog Scale (VAS) for pain in the lumbar region that extends to the lower limbs.

The survey collected demographic information such as age, gender, weight, height (expressed as body mass index or BMI), employment status, regular physical activity, smoking history, and comorbidities. The clinical factors considered were time of onset of symptoms, side affected, and presence of motor or sensory deficits.

The RedCAP system was used for storage and confidentiality of the acquired data, with exclusive access granted to the research team following verification and approval by the São Paulo Hospital Research Ethics Committee (CoEP).

### Statistical analysis

Statistical analysis was conducted using SPSS Statistics software program, based on the variables exported from the RedCAP database.

Categorical and continuous variables were analyzed by calculating the frequency and the mean standard deviations, respectively. The chi-squared test and Fisher’s exact test were applied for statistical evaluation in the contingency table and the Student’s t-test for independent samples.

To determine the correlation between demographic and clinical factors and dysfunction observed during the initial consultation (assessed by the Oswestry scale), participants were split into two groups: mild to severe dysfunction (ODI < 60), which does not interfere with daily activities, and incapacitating dysfunction (ODI ≥ 60), which requires assistance to perform these activities. Sociodemographic data between individuals in both groups were compared. Statistical significance was determined at p < 0.05 (>95% confidence interval).

Correlation tests such as Pearson’s Coefficient and Spearman’s Coefficient were performed to analyze the strength of the association for statistically significant differences.

## RESULTS

From July to December 2020, 42 patients received treatment for a clinical condition consistent with acute sciatica, representing roughly 1.4% of all orthopedic consultations provided by this service during that period. From these patients, 19 (45.2%) were male and 23 (54.8%) were female. The mean age of the participants was 44.2 years, with a standard deviation of 16.35 years. We found no significant difference between sexes. Regarding ethnicity and skin color, 15 patients identified as Mixed-race (37.5%), 13 (32.5%) as White, 11 (27.5%) as Black, and three participants were unable to define.

The mean body mass index (BMI) was 28, with a standard deviation of 5.49. When stratified by sex, the mean for male patients was 27.03 with a standard deviation of 4.71, whereas for female patients it was 29.73 with a standard deviation of 5.66. However, we found no statistically significant difference between the two (p = 0.11). Regarding habits, 10 (23.8%) patients reported regularly using tobacco, and 36 (85.7%) participants reported not engaging in regular physical activity (at least three times a week). Of the participants who did exercise regularly, four reported running or walking and two engaged in gym activities.

Concerning employment status, 34 patients (81.91%) held formal jobs with contracts, four (9.5%) were unemployed (either seeking work or working informally), and four (9.5%) were already retired. Of those who retired, two (50.0%) were due to length of service and two (50.0%) due to previous pathologies/incidents.

Table 1 summarizes the sociodemographic variables.

**Table 1:** Summary of the sociodemographic data obtained in the interview.

		N	% of N	Mean	Standard deviation
Sex	Male	19	45.2		
	Female	23	54.8		
Age	18-20	2	4.8	44.98	15.31
	21-40	15	35.7		
	41-60	20	47.6		
	> 60	5	11.9		
Skin color	White	13	32.5		
	Mixed race	15	37.5		
	Indigenous people	0	0.0		
	Yellow	1	2.5		
	Black	11	27.5		
Body Mass Index	Underweight	0	0.0	28.51	5.49
	Normal	9	21.4		
	Overweight	21	50.0		
	Grade I obesity	7	16.7		
	Grade II obesity	3	7.1		
	Grade III obesity	2	4.8		
Professional status	Active	34	81.0		
	Unemployed	4	9.5		
	Social security	0	0.0		
	Retired	4	9.5		
Active smoking	Yes	10	23.8		
	No	32	76.2		
Regular physical activity?	No	36	85.7		
	Yes	6	14.3		
Total		42	100		

Considering the clinical history of the current pathology, 9 (21.4%) patients reported that the symptoms started less than 7 days ago, whereas 22 (52.4%) patients reported that the onset occurred 7 to 30 days ago, and 11 (26.2%) patients sought medical attention from 30 days to 3 months after the onset of the current condition. For 19 patients (45.2%), acute sciatic pain was a new occurrence, whereas 23 participants (54.2%) had reported a similar event for more than one year but were asymptomatic.

The physical examination identified the presence of motor deficits (considering the global strength scale proposed by the Medical Research Council) in seven patients (35.3%). Among these, four patients had more than one affected root, of which six (85.2%) had a deficit in the L5 lumbar root, three (42.9%) in the L4 root, and two (28.6%) had a clinically evident reduction in motor strength in the S1 root. Sensory deficits were noted in 16 participants (38.1%). Hypoesthesia was identified in the L4 dermatome of nine participants (56.3%), the L5 dermatome of eight (50%), and S1 in six (37.5%).

**Figure 1:**
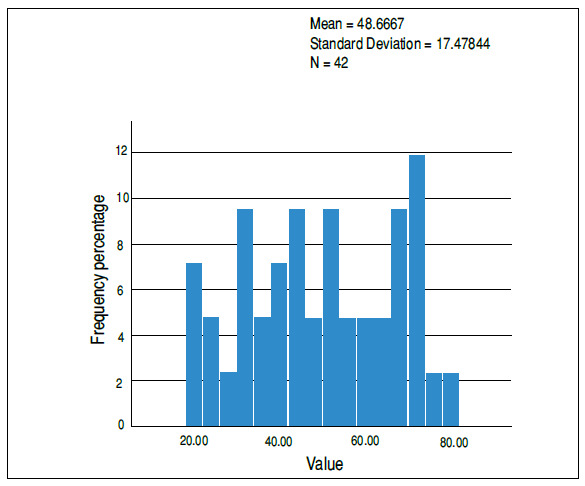
Histogram representing the distribution of values obtained with the application of the ODI.

Regarding the assessment of function using the Oswestry scale, it was found that the sample followed a normal pattern after applying the Shapiro-Wilk test. The mean value was 49 points, with a SD of 17.48 (Figure 1). According to sex, the mean for males was 49.89, with a SD of 15.45, and the mean for females was 47.65, with a SD of 18.57, showing no statistical difference (p = 0.68). When stratified by severity of dysfunction, we found a statistically normal distribution, with 15 (35.7%) patients showing a degree of dysfunction considered mild/moderate (ODI up to 40 points), 14 considered severe (ODI from 41 to 60 points), and 13 with disabling dysfunction (ODI greater than 60 points).

The intensity of pain at the initial consultation was assessed using the VAS, and the mean value found for low back pain was 7.11, with a SD of 2.49, whereas for radiating low back pain it was 6.71, with a SD of 2.28. Table 2 summarizes the data obtained by clinical evaluation.

**Table 2:** Summary of the data obtained by the physical examination and application of the Oswestry and VAS forms.

		N	% of sample
Time of onset of symptoms	From 0 to 7 days	9	21.4
	7 days to 1 month	22	52.4
	From 1 to 3 months	11	26.2
Motor deficit	Yes	7	16.7
	No	35	83.3
Similar previous episodes	Yes	23	54.8
	No	19	45.2
Sensory deficit	Yes	16	38.1
	No	26	61.9
ODI value (in categories)	Mild/moderate	15	35.7
	Severe	14	33.3
	Crippling	13	31.0
VAS for radiating low back pain	Mild/moderate	6	14.3
	Severe	18	42.9
	Very severe	18	42.9
VAS for axial low back pain	Mild/moderate	7	16.7
	Severe	14	33.3
	Very severe	21	50.0

Furthermore, we investigated the relationship between sociodemographic variables and degree of dysfunction. A cut-off point of 60 was used since it is considered a determining factor for functional incapacity. To compare the prevalence of variables, contingency tables (Tables 3 and 4) were constructed. A statistically significant difference (p = 0.046) was found between the groups regarding sociodemographic variables, with no significant differences between sex, age group, BMI, physical activity, and smoking habit.

**Table 3:** Contingency table for comparison of sociodemographic variables according to the severity of the dysfunction.

		Non-disabling (< 60) 29 (69.0%)	Crippling (≥ 60) 13 (31.0%)	Total - 42 (100%)	Significance (p-value)
Sex	Male	14 (33.3%)	5 (11,9%)	19 (45.2%)	0.401
	Female	15 (35.7%)	8 (19.0%)	23 (54.8%)	
Age (in categories)	≤ 40 years	14 (33.3%)	3 (7.1%)	17 (40.5%)	0.115
	> 40 years	15 (35.7%)	10 (28.3%)	25 (59.5%)	
Body mass index (in categories)	Normal	7 (16.7%)	2 (4.8%)	9 (21.4%)	0.421
	Overweight/obesity	22 (52.4%)	11 (26.2%)	33 (78.6%)	
Active smoking	Yes	6 (14.3%)	4 (9.5%)	10 (23.8%)	0.367
	No	23 (54.8%)	9 (21.4%)	32 (76.2%)	
Regular physical activity (at least 3x/week)	No	25 (59.5%)	11 (26.2%)	36 (85.7%)	0.615
	Yes	4 (9.5%)	2 (4.8%)	6 (14.3%)	
Professional activity	Formal employment	26 (61.9%)	8 (19.0%)	34 (81.0%)	0.046
	Inactive (unemployment, retirement)	3 (7.1%)	5 (11,9%)	8 (19.0%)	

**Table 4:** Contingency table for comparison of clinical variables according to the severity of the dysfunction.

		Non-disabling (< 60) 29 (69.0%)	Crippling (≥ 60) 13 (31.0%)	Total - 42 (100%)	Significance (p-value)
Similar previous episodes - N (%)	Yes	16 (38.1%)	7 (16.75%)	23 (54.8%)	0.599
	No	13 (31.0%)	6 (14.3%)	19 (45.2%)	
Time of onset of symptoms - N (%)	From 0 to 7 days	7 (16.7%)	2 (4.8%)	9 (21.4%)	0.336
	7 days to 1 month	13 (31.0%)	9 (21.4%)	22 (52.4%)	
	From 1 to 3 months	9 (21.4%)	2 (4.8%)	11 (26.2%)	
Motor deficit - N (%)	Yes	2 (4.8%)	5 (11,9%)	7 (16.7%)	0.021
	No	27 (64.3%)	8 (19.0%)	35 (83.3%)	
Sensory deficit - N (%)	Yes	9 (21.4%)	7 (16.7%)	16 (38.1%)	0.144
	No	20 (47.6%)	6 (14.3%)	26 (61.9%)	
VAS for axial low back pain - N (%)	Mild/Moderate (0 to 4)	5 (11,9%)	2 (4.8%)	7 (16.7%)	0.199
	Severe (5 to 7)	12 (28.6%)	2 (4.8%)	14 (33.3%)	
	Very severe (8 to 10)	12 (28.6%)	9 (21.4%)	21 (50.0%)	
VAS for radiating low back pain - N (%)	Mild/Moderate (0 to 4)	6 (14.3%)	0 (0.0%)	6 (14.3%)	0.043
	Severe (5 to 7)	13 (31.0%)	5 (11,9%)	18 (42.9%)	
	Very severe (8 to 10)	10 (23.8%)	8 (19.0%)	18 (42.9%)	

Using the same cut-off value for dysfunction, a contingency table was constructed to compare both groups regarding clinical data. A statistically significant difference was observed between them in terms of the presence of motor deficits (p = 0.021) and the intensity of pain symptoms radiating low back pain measured by the VAS (p = 0.043).

Finally, measures of association were applied, such as Pearson’s Coefficient of Contingency and Spearman’s correlation. The values indicated a moderate association between professional activity (R = 0.33) and intensity of radiating low back pain (R = 0.77), along with the presence of disabling dysfunction secondary to acute sciatica and motor deficit (R = 0.778).

## DISCUSSION

When acute low back pain radiates to the lower limbs, it is referred to as radicular or radiculopathy pain, which is a primary cause of dysfunction in individuals aged 20 to 60 years.^10^ This leads to significant direct and indirect economic impacts, estimated to be around $100 billion annually in the United States.^11^ Lumbar disc herniation is the most prevalent associated pathology. However, degenerative changes including facet hypertrophy, synovial cysts, as well as tumorous and infectious causes may also elicit this clinical presentation.

To the best of our knowledge, no studies in the literature have evaluated the clinical and demographic characteristics of patients with acute radicular pain in the Brazilian population. A study by Gotfryd et al.^12^ conducted an epidemiological assessment of patients with acute low back pain based on a sample of the Brazilian population. Our study differs from that one by several factors. We highlight that both studies examined a population sample with lumbar spinal pathologies with acute onset (less than 3 months) of the same nationality. However, they differed in the socioeconomic status of the participants. The first study was conducted in a high-cost private hospital, whereas our study was conducted in a referral service of the Brazilian Unified Health System. Furthermore, our study focused on individuals with a clinical picture consistent with acute radiculopathy, whereas the previous study emphasized participants with predominantly axial pain.

The sample of 41 patients evaluated in this study showed a normal distribution concerning sociodemographic characteristics (Table 1). The patient demographics, comprising sex (with a slight female predominance of 54.8%), age (mean 44 ± 15.3 years), and skin color align with data obtained from epidemiological studies among diverse populations.^13^ The majority of patients (78.1%) had a BMI exceeding normal values (> 24.9), with a sample mean of 28.5 ± 5.4 and 28.6% of individuals indicating obesity (BMI ≥ 30). These figures are also consistent with the distribution in the general Brazilian population, whose estimated prevalence of overweight and/or obesity is 61.7% according to the most recent IBGE data.^14^ Furthermore, in a meta-analysis that included 26 studies, published in 2013, Shiri et al.^15^ identified a statistically significant correlation between overweight/obesity and the prevalence of sciatica in the general population. Furthermore, a positive correlation was found between BMI above the values considered normal and hospitalization, as well as increased risk of hospitalization and surgeries related to the clinical condition.

In another meta-analysis published by the same author,^16^ in 2015, with around 28 articles, active smoking was identified as a moderate risk factor (OR = 1.64) for the development of acute radicular pain. In our sample, the prevalence of active smokers was estimated at 23.8%. Considering that approximately 12.8% of the Brazilian population report using tobacco products, according to data from the Brazilian National Health Survey, the prevalence among individuals with radiating low back pain evaluated in this study is significantly higher than in the general population.

Regarding the clinical characteristics of this group of patients, the mean ODI value was 48.6 ± 17.47, with a median of 49.0, which is considered severe dysfunction according to the scale. This value is substantially higher than that found in most comparable literature that, despite evaluating different outcomes, involved a sample of patients with radicular pain. Meyer et al.,^17^ in a research project aimed at comparing the results of endoscopic surgery with microdiscectomy for lumbar disc herniation, included a sample of 47 participants whose mean ODI value was 29.0 ± 8.8. In another study series, which aimed at the efficacy of anesthetic transforaminal injection for the treatment of acute radicular pain,^18^ with a sample of 61 participants, the mean value was 40.85 ± 5.36. It is important to note, however, that both studies involved patients whose sciatica pain persisted even after clinical treatment, unlike our study. When different populations are considered by a group of researchers from other countries, the reported mean values are also considerably lower, ranging from 30 ± 13.2 in a study by Iversen et al.^19^ to 42.4 (ranging from 14 to 80) in an article published by Kennedy et al.^20^. Furthermore, Konstatinou et al^21^ compared the functional impact of acute radicular pain with that of low back pain alone and found that low back pain with radiating pain was statistically more disabling than those without associated radicular pain. In that series, the authors demonstrated a mean baseline ODI value for patients with sciatica of about 49.1 (6-86), similar to our research.

One of the main causes associated with this higher rate of dysfunction in initial care can undoubtedly be attributed to the prevailing social context at the time of this research. Patients were recruited for this study during the COVID-19 pandemic, which began in Brazil at the end of February and continued until the publication date of this study. In the absence of an effective treatment to slow down or prevent the spread of Sars-CoV-2, the main prevention policies were the use of masks and social distancing. Furthermore, public authorities have recommended that the general public avoid hospital environments due to the higher risk of contagion in these locations, except in the most serious cases.^22^

As a result, it is possible that most people who had milder acute radicular pain did not seek medical attention due to public health guidelines aimed at reducing COVID-19 transmission. Therefore, it is possible that “indirect selection” of patients occurred, that is, those with greater dysfunction were the ones who opted to be evaluated in a more complex hospital, which is also a regional reference in COVID-19 treatment, thus increasing the baseline value of the dysfunction index evaluated by the ODI.

To compare clinical and demographic variables among individuals with incapacitating dysfunction (ODI ≥ 60 points) and those with mild to severe dysfunction (ODI < 60 points), we designed a contingency table that compares both groups (Table 3). We found that the group classified with incapacitating dysfunction included 13 individuals (31.0%), whereas those with less severe dysfunction corresponded to 29 individuals (69.0%). Performing the prevalence ratio between the two, we observed a statistically significant difference in relation to professional activity (p = 0.046), motor deficit (p = 0.021), and intensity of radiated pain (p = 0.043).

When assessing variations in professional activity, a higher prevalence of inactive patients, either due to unemployment or retirement, was found in the group with greater dysfunction compared with those with an ODI < 60 points, who were mostly formally employed. Among the possible explanations, employee payment and benefits are highlighted. In 2000, Atlas et al.^23^ concluded that patients receiving some kind of work compensation had a higher risk of exhibiting greater dysfunction at initial care than those who were professionally active. The same researchers, using data obtained from the Spine Patient Outcomes Research Trial (SPORT)^24^ demonstrated statistically significant differences related to outcomes after conservative and surgical treatment between individuals who were receiving social security compensation and those who were not. Thus, evaluating potential secondary benefits associated with the presented dysfunction is crucial in determining the appropriate treatment.

Another possible explanation, which has become even more important during the context of economic instability due to the COVID-19 pandemic, is related to the deterioration of mental health with the appearance of symptoms of anxiety, fear, and distress related to financial difficulties, which are more prevalent in professionally inactive individuals. The association between the perception of acute sciatic pain, both in terms of intensity and dysfunction, and mental health is well established, as is the prognosis and evolution of the clinical picture.^25^

Finally, a statistically significant higher prevalence of objective motor deficits (muscle strength ≤ 3 on the MRC scale) was identified among those with a degree of dysfunction considered crippling. The correlation coefficient analysis identified a strong association between the severity of the dysfunction and the presence of reduced motor strength. The mean ODI for patients with motor deficits (7 patients, approximately 16%) was 64.5 ± 12.6, whereas the mean ODI for patients without deficits (36 patients, approximately 83.66%) was 45.4 ± 16.6, with a statistically significant difference (p = 0.007) between the groups. These findings agree with the case series described by Falavigna et al.^26^ whose aim was to assess whether the presence of motor deficits influenced post-operative outcomes in patients with herniated lumbar discs. In this situation, the authors found a slight variance in the mean ODI score between the groups, which was statistically significant but did not meet the minimal clinically significant difference (10 points) recognized for this measure. This suggests that although the difference was statistically significant, it was not clinically relevant.

Our opinion is that the sample in this study displays distinct characteristics from the 2014 article since it only included patients who sought emergency care due to recent onset of symptoms. In contrast, the previous article focused on patients whose conservative treatment had failed, receiving surgical indication. Thus, it is possible that patients who have had the deficit for a longer period have already adapted their usual and professional routines to the limitation in motor strength, and, therefore, the functional impact has diminished over time. This opinion is strengthened by an article published by Stienen et al.^27^ in 2020, according to which quality of life forms, including the ODI, tend to underestimate the impact of motor deficits on the function of patients with spinal pathologies.

This study contains some limitations. The main reason for this is the exceptional social context caused by the COVID-19 pandemic, which, combined with the recommendations for social isolation by health authorities, has affected the treatment provided by healthcare services for several diseases. Thus, the demographic and social portrait of patients with acute sciatic pain reproduced by this study may not represent the pattern observed in periods of normality. Research conducted in the post-pandemic period could more accurately reflect the manifestation of this pathology in the Brazilian population and also allow assessment of the impact of the pandemic on the outcome of these patients.

Furthermore, since this is a cross-sectional observational study with a single-center sample, the analysis of causal factors associated with dysfunction in this group of patients is limited. Longitudinal studies are needed to assess whether the variables that showed statistically significant differences in relation to the proportions have a causal relationship with the severity measured by the ODI. Since this is a sub-project of another protocol developed by the same group of researchers, whose primary outcome is the clinical and functional response after treatment with oral corticosteroids, patients with comorbidities or contraindications to the use of this class of medication, especially those with type 2 diabetes mellitus, were not included.

## CONCLUSION

This study aimed to analyze the profile of Brazilian patients who seek emergency medical attention due to acute radicular pain, with onset of up to three months. We found that these patients suffer from more significant functional impairment compared with samples from similar series conducted in other countries. Around 30% of the individuals had functional impairment considered to be crippling, and the presence of motor deficits, intensity of radiating pain, and professional inactivity was statistically higher in this group than in the others, suggesting that these variables may influence the perception of symptoms.

However, we highlight that the evaluation was conducted during the COVID-19 pandemic, and the social isolation measures recommended during that period may have resulted in only patients with more severe clinical conditions seeking medical care, given the risks associated with SARS-CoV-2 infection. Future longitudinal studies should be conducted to assess the impact of the pandemic on the management of this group of patients.
